# Evaluation of Attention Switching and Duration of Electronic Inbox Work Among Primary Care Physicians

**DOI:** 10.1001/jamanetworkopen.2020.31856

**Published:** 2021-01-21

**Authors:** Tracy A. Lieu, E. Margaret Warton, Jeffrey A. East, Mark F. Moeller, Stephanie Prausnitz, Manuel Ballesca, Gloria Mark, Fatema Akbar, Sameer Awsare, Yi-Fen Irene Chen, Mary E. Reed

**Affiliations:** 1 Division of Research, Kaiser Permanente Northern California, Oakland; 2 The Permanente Medical Group, Oakland, California; 3 Department of Adult and Family Medicine, Kaiser Permanente, Richmond, California; 4 Department of Adult and Family Medicine, Kaiser Permanente, San Rafael, California; 5 Department of Adult and Family Medicine, Kaiser Permanente, Napa, California; 6 Department of Informatics, Donald Bren School of Information and Computer Sciences, University of California, Irvine

## Abstract

**Importance:**

Primary care physicians (PCPs) report multitasking during workdays while processing electronic inbox messages, but scant systematic information exists on attention switching and its correlates in the health care setting.

**Objectives:**

To describe PCPs’ frequency of attention switching associated with electronic inbox work, identify potentially modifiable factors associated with attention switching and inbox work duration, and compare the relative association of attention switching and other factors with inbox work duration.

**Design, Setting, and Participants:**

This cross-sectional study of the work of 1275 PCPs in an integrated group serving 4.5 million patients used electronic health record (EHR) access logs from March 1 to 31, 2018, to evaluate PCPs’ frequency of attention switching. Statistical analysis was performed from October 15, 2018, to August 28, 2020.

**Main Outcomes and Measures:**

Attention switching was defined as switching between the electronic inbox, other EHR work, and non-EHR periods. Inbox work duration included minutes spent on electronic inbox message views and related EHR tasks. Multivariable models controlled for the exposures.

**Results:**

The 1275 PCPs studied (721 women [56.5%]; mean [SD] age, 45.9 [8.5] years) had a mean (SD) of 9.0 (7.6) years of experience with the medical group and received a mean (SD) of 332.6 (148.3) (interquartile range, 252-418) new inbox messages weekly. On workdays, PCPs made a mean (SD) of 79.4 (21.8) attention switches associated with inbox work and did a mean (SD) 64.2 (18.7) minutes of inbox work over the course of 24 hours on workdays. In the model for attention switching, each additional patient secure message beyond the reference value was associated with 0.289 (95% CI, 0.217-0.362) additional switches, each additional results message was associated with 0.203 (95% CI, 0.127-0.278) additional switches, each additional request message was associated with 0.190 (95% CI, 0.124-0.257) additional switches, and each additional administrative message was associated with 0.262 (95% CI, 0.166-0.358) additional switches. Having a panel (a list of patients assigned to a primary care team) with more elderly patients (0.144 switches per percentage increase [95% CI, 0.009-0.278]) and higher inbox work duration (0.468 switches per additional minute of inbox work [95% CI, 0.411-0.524]) were also associated with higher attention switching involving the inbox. In the model for inbox work duration, each additional patient secure message beyond the reference value was associated with 0.151 (95% CI, 0.085-0.217) additional minutes, each additional results message was associated with 0.338 (95% CI, 0.272-0.404) additional minutes, each additional request message was associated with 0.101 (95% CI, 0.041-0.161) additional minutes, and each additional administrative message was associated with 0.179 (95% CI, 0.093-0.265) additional minutes. A higher percentage of the panel’s patients initiating messages (0.386 minutes per percentage increase [95% CI, 0.026-0.745]) and attention switches (0.373 minutes per switch [95% CI, 0.328-0.419]) were also associated with higher inbox work duration. In addition, working at a medical center where all PCPs had high inbox work duration was independently associated with high or low inbox work duration.

**Conclusions and Relevance:**

This study suggests that PCPs make frequent attention switches during workdays while processing electronic inbox messages. Message quantity was associated with both attention switching and inbox work duration. Physician and patient panel characteristics had less association with attention switching and inbox work duration. Assisting PCPs with message quantity might help modulate both attention switching and inbox work duration.

## Key Points

**Question:**

Among primary care physicians (PCPs), how frequent is attention switching associated with the electronic inbox work during workdays, and what factors are associated with attention switching and duration of inbox work?

**Findings:**

Among 1275 PCPs studied in this cross-sectional study, PCPs switched attention to or from the inbox a mean of 79 times and spent 64 minutes doing inbox work on workdays. Message quantity was a dominant factor associated with attention switching and inbox work duration.

**Meaning:**

This study suggests that PCPs make frequent attention switches to and from the inbox while working, and interventions to assist them with message quantity could modulate both attention switching and inbox work duration.

## Teaser

This cross-sectional study examines primary care physicians’ frequency of attention switching associated with electronic inbox work and identifies potentially modifiable factors associated with attention switching and inbox work duration.

## Introduction

Primary care physicians (PCPs) have identified the inbox within the electronic health record (EHR) as a source of work-related stress.^1,2,3,4^ One possible reason is that it adds to the multiple demands that compete for PCPs’ attention during the workday. Given that PCPs spend large portions of their time using EHRs,^5,6,7,8^ electronic inbox work could be associated with many switches in attention when processing messages amid patient visits and other clinical responsibilities.

Attention switching during medical work, whether due to external interruptions or internal volition, is of interest because it causes cognitive burden and may be associated with errors.^9,10,11,12,13^ In studies outside medicine, email management has been associated with multitasking and stress.^14,15^ Multitasking with email as measured by switching of computer screens is associated with inefficiency and feelings of diminished productivity.^16,17^ However, to our knowledge, attention switching with the electronic inbox has not previously been studied in medicine.

We designed this study to address current knowledge gaps about the characteristics of electronic inbox work and to identify potential strategies to support PCPs. Our aims were to describe PCPs’ frequency of attention switching associated with electronic inbox work, identify potentially modifiable factors associated with attention switching and inbox work duration, and compare the relative association of attention switching and other factors with inbox work duration.

## Methods

### Design and Study Setting

In this retrospective cross-sectional study, we evaluated the electronic inbox work patterns of full-time PCPs in the largest medical group in the United States. We summarized EHR access log data generated by internists and family medicine physicians of The Permanente Medical Group, which has 9200 physicians and is part of Kaiser Permanente Northern California, an integrated system serving 4.5 million members in 21 hospital-based medical centers. Primary care physicians are grouped in clinic modules across 60 facilities.

Since 2008, Kaiser Permanente Northern California has used a comprehensive EHR (Epic) that integrates inpatient, emergency, and outpatient care. The electronic inbox receives messages patients sent via a portal website or patient-facing mobile applications, as well as messages from other physicians, clinical staff, the pharmacy, laboratory, and other departments. Physicians can access inboxes on computers or mobile devices and are expected to reply to patient messages within 2 business days. At the time of this study, patient messages were sent directly to physicians’ inboxes. Most medical center departments encouraged physicians to have their medical assistants help with specific types of messages. This study was approved by the Kaiser Permanente Northern California Institutional Review Board with a waiver of informed consent. This study followed the Strengthening the Reporting of Observational Studies in Epidemiology (STROBE) reporting guideline.

### Data Collection and Analysis

This study included all internists and family medicine physicians who spent at least 28 hours a week (0.7 full-time equivalents) in direct patient care, because they represent typical clinicians. For these physicians, computerized data from the Epic access log from March 1 to 31, 2018, were summarized into variables that described specific physician actions. We selected March because it has medium work volume. We defined full workdays as having 2 half-day shifts with at least 1 in-person, telephone, or video appointment scheduled in each half-day shift. We collected computerized data on PCP characteristics, clinical practice measures, and patient panel (a list of patients assigned to a primary care team) characteristics, including propensity to initiate secure messages.

We combined and summarized raw source data from the access log and access workflow tables, along with the detail data for each source (rather than using Epic’s presummarized data reports). We generated time segments that assigned each second during the month to a specific activity. The following 3 general work contexts were defined: (1) inbox work, (2) other EHR work, and (3) non-EHR time. Inbox work periods started when a PCP opened an inbox message and ended when the PCP completed activities associated with inbox messages and switched to another activity. For example, opening a patient’s secure message followed by a review of the patient’s medical record, placing an order, and returning to the inbox to reply to the patient was not counted as an attention switch, because all actions occurred in the context of processing the patient’s secure message. Other EHR work included all actions not occurring after inbox message access, such as reviewing patient medical records, entering orders, or writing medical record notes. Non-EHR time was defined as a period that elapsed with no actions in the EHR.

For most actions, if 45 seconds or more elapsed with no EHR activity prior to an action, the preceding period was classified as non-EHR time. This criterion was based on operational reporting standards and our review of the frequency distributions of time gaps in preliminary data. In selected situations, we used a higher criterion to capture actions that often required more than 45 seconds. For example, the action of sending a reply to a patient’s message was often preceded by more than 45 seconds while the PCP typed the reply. As the Epic access log detects only mouse clicks initiating or completing an action, it did not record time spent typing the reply. Thus, for the time period prior to sending a reply to a patient’s message, we allowed up to 120 seconds, enabling us to capture and count most periods spent typing messages.

Attention switching was defined as switching from one work context to another. A PCP doing inbox work switching to other EHR work was counted as a switch from inbox to other EHR work. If a PCP was inactive on the EHR and then opened an inbox message, this was counted as a switch from non-EHR work to inbox work. For this analysis, switches between the inbox and other parts of the EHR while working on inbox messages were not counted as context switches. Inbox messages were initially assigned to 1 of 63 specific types by the EHR system, which we grouped into the following 4 categories: patient-initiated secure messages, results (including laboratory tests and imaging), requests (mostly patient call center requests, refill approvals, and order cosigns), and informational or administrative (such as messages on which the PCP was copied that contained medical records).

For selected analyses, we excluded outliers based on our knowledge of local practice patterns. For example, in some departments, a designated physician covers for other physicians who are on vacation and appears to spend an entire shift performing inbox work. Thus, in analyses of inbox work quantity and duration, we excluded days where physicians viewed more than 250 messages based on data inspection and clinical input, inferring that they were covering inbox work for their department.

This study’s cross-sectional design could not elucidate whether more attention switching caused longer inbox work duration or whether having a longer duration of inbox work simply created more opportunities for attention switching. To address this, we created a variable that reflected a physician’s propensity to complete each message in one sitting, formulated to be independent of the amount of time spent in the inbox. This variable was the number of work segments per unique message, where a segment was a discrete period of work on a message. For example, a message that was opened on 3 separate occasions resulted in 3 segments. We hypothesized that physicians who opened a single message multiple times would have higher attention switching and higher inbox work duration.

### Statistical Analysis

Statistical analysis was performed from October 15, 2018, to August 28, 2020. In preliminary analyses, we evaluated each potential correlate of the 2 key outcomes (attention switching and inbox work duration) via bivariate models. For each outcome, we then created a series of 6 forced-entry multivariable linear regression models to evaluate the independent association of each factor while controlling for other variables, with the statistical significance level prespecified at *P* < .05. Statistical tests were 2-sided. The modeling sequence followed a conceptual model (eFigure in the Supplement) and included physician characteristics, patient panel and medical center–level variables, patient panel propensity to initiate secure messages, message quantity variables, work segments per unique message, and attention switching or inbox work duration. Analyses were conducted in SAS, version 9.4 (SAS Institute Inc).

## Results

### Study Population and Outcomes

Among the 1275 PCPs in the study, the mean (SD) age was 45.9 (8.5) years; 721 were female (56.5%), 716 were internists (56.2%), and 559 were family medicine physicians (43.8%), and they had a mean (SD) of 9.0 (7.6) years of experience with the medical group (Table 1). Among the PCPs’ patient panels, the mean (SD) percentage of patients initiating a secure message during the 31-day study period was 11.8% (3.2%).

**Table 1.** Characteristics of the Study Population of Primary Care Physicians

Characteristic	Primary care physicians, No. (%) (N = 1275)
Specialty	
Internal medicine	716 (56.2)
Family medicine	559 (43.8)
Age, mean (SD), y	45.9 (8.5)
Female	721 (56.5)
Race/ethnicity^a^	
Asian	805 (63.1)
Black	48 (3.8)
Latino	93 (7.3)
White	314 (24.6)
Experience	
Years since medical school graduation, mean (SD)	19.1 (9.3)
Years with the medical group, mean (SD)	9.0 (7.6)
FTE worked, mean (SD)^b^	0.85 (0.10)
Patient panel characteristics^c^	
Patients in panel aged ≥65 y, mean (SD), %	18.5 (9.8)
Female patients in panel, mean (SD) %	51.8 (18.1)
Patients in panel who initiated a portal secure message during the study period, mean (SD), %^d^	11.8 (3.2)

Abbreviation: FTE, full-time equivalents.

^a^ Fifteen physicians (1.2%) had other or unknown race/ethnicity.

^b^ Physicians working 0.7 or more FTE in clinical practice were eligible for inclusion in the study.

^c^ Other patient panel characteristics tested in preliminary bivariate analyses included mean and median age and percentage of panel that was White, Black, Asian, Hispanic, and other race/ethnicity.

^d^ The study period covered from March 1 to 31, 2018.

Physicians received a mean (SD) of 332.6 (148.3) (interquartile range, 252-418) new messages per week. These messages resulted in 585 message views per week, as messages could be viewed more than once. Of the message views, 133 (22.7%) were patient-initiated secure messages; 175 (29.9%) were results from laboratory tests, imaging, or other procedures; 164 (28.0%) were requests from the call center, pharmacy, or other clinicians; and 114 (19.5%) were administrative or informational messages such as messages on which the PCP was copied that contained medical records.

The analysis of full workdays included 19 395 physician-days, during which physicians made a daily mean (SD) of 79.4 (21.8) attention switches associated with inbox work (Table 2). Slightly more than half the switches into inbox work were from non-EHR periods (mean [SD], 21.1 [6.4]); the rest were from other EHR work (mean [SD], 18.6 [7.1]).

**Table 2.** Attention Switching and Inbox Work Duration Among Primary Care Physicians

Attention switches	Attention switches during full workdays^a^			*P* value^b^
	All physicians	Physicians in lowest quartile for attention switching	Physicians in highest quartile for attention switching	
Switches into inbox work, mean (SD), No.				
From other EHR work	18.6 (7.1)	16.4 (6.4)	21.1 (7.7)	<.001
From non-EHR work	21.1 (6.4)	19.9 (6.1)	22.0 (7.0)	<.001
Switches out of inbox work				
To other EHR work	16.0 (6.8)	14.3 (5.9)	18.1 (7.8)	<.001
To non-EHR work	23.7 (7.0)	22.0 (6.6)	25.0 (7.6)	<.001
Total No. of switches involving inbox, mean (SD)	79.4 (21.8)	72.5 (21.1)	86.2 (22.7)	<.001
Duration of inbox work on full workdays, mean (SD), min^a^				
				
During work hours	40.6 (14.0)	38.6 (14.5)	40.2 (13.6)	.48
Outside work hours^c^	23.6 (12.8)	19.6 (11.2)	28.2 (14.6)	<.001
Total No. (24 h, including the full workday), mean (SD)	64.2 (18.7)	58.1 (19.0)	68.4 (18.7)	<.001

Abbreviation: EHR, electronic health record.

^a^ Workdays included 24 hours on weekdays when the physician had both morning and afternoon clinical work, excluding days when physicians appeared to be doing entire shifts covering others’ inboxes.

^b^ For the comparison of the first vs fourth quartile for attention switching, using analysis of variance and a Tukey-Kramer adjustment for multiple comparisons.

^c^ Work hours were defined as scheduled outpatient clinic hours (8:30 am-12:30 pm and 1:30-5:30 pm), not including the lunch hour.

The mean (SD) duration of inbox work on full workdays was 64.2 (18.7) minutes. As shown in Table 2, physicians in the highest quartile for attention switching had a higher mean (SD) duration of inbox work (68.4 [18.7] minutes) than those in the lowest quartile (58.1 [19.0] minutes; *P* < .001).

### Correlates of Attention Switching

In the final multivariable model for attention switching, higher quantities of inbox messages of all 4 types were associated with more switching per workday (eTable 1 in the Supplement). Each additional patient secure message beyond the reference value was associated with 0.289 (95% CI, 0.217-0.362) additional switches, each additional results message was associated with 0.203 (95% CI, 0.127-0.278) additional switches, each additional request message was associated with 0.190 (95% CI, 0.124-0.257) additional switches, and each additional administrative message was associated with 0.262 (95% CI, 0.166-0.358) additional switches. In addition, being a male physician (2.798 switches per workday [95% CI, 1.179-4.416]), being a younger physician (0.124 switches per year of age [95% CI, 0.003-0.245]), having a larger patient panel (0.003 switches per patient [95% CI, 0.000-0.005]), having a higher percentage of patients aged 65 years or older (0.144 switches per percentage increase [95% CI, 0.009-0.278]), having more discrete work segments per message (0.129 switches per work segment [95% CI, 0.097-0.162]), and having longer inbox work duration (0.468 switches per additional minute of inbox work [95% CI, 0.411-0.524]) were associated with more attention switching. This model explained a large amount of variability in the outcome (*R*^2^ = 0.68).

Table 3 illustrates the association of these factors with the estimated number of attention switches per workday. To estimate the relative effect size of each factor, we changed each measure from lower to higher values (for continuous variables, the 25th and 75th percentiles) while holding all other variables at their reference values. Variability in message quantity (which resulted in an increase of 3 to 5 switches, depending on message type) and inbox work duration (resulting in an increase of 12 switches) accounted for the largest changes in attention switching. In contrast, physician and patient panel factors accounted for less of the variability in attention switching.

**Table 3.** Variation Among Primary Care Physicians in Attention Switching Involving the Electronic Inbox When Key Factors Were Varied From Low and High Values

Factor^a^	Value			Estimated No. of attention switches^c^		Additional No. of attention switches as factor is varied from 25th to 75th percentile
	Reference^b^	25th Percentile	75th Percentile	25th Percentile	75th Percentile	
Message quantity, mean No. per workday						
Patient messages	29	19	37	79	84	5
Results	35	26	43	80	83	3
Requests	36	24	46	80	84	4
Informational	23	17	28	80	83	3
Panel characteristics						
Patients >65 y, %	17	11	24	81	83	2
Other factors						
Inbox work duration, mean daily min	64	51	76	76	87	12
Work segments per 100 unique messages, mean^d^	176	152	191	79	84	5

^a^ Other factors in the final multivariable model included physician age (2 fewer switches as value was varied from the 25th to the 75th percentile), physician sex (3 fewer switches among female vs male PCPs), and panel size (1 fewer switch per day as value was varied from the 25th to the 75th percentile). See eTable 1 in the Supplement for the detailed estimates, 95% CIs, and *P* values from this model.

^b^ Reference value used in final multivariable model. When all values were set at the reference value, the physician was estimated to make 82 attention switches owing to inbox work in a full workday.

^c^ Estimated number is based on adjusted means from the final multivariable model. All variables in the table and in footnote a were significant at *P* < .05.

^d^ A unique message is a message opened for the first time during the month.

### Correlates of Inbox Work Duration

In preliminary analyses, we found that inbox work duration was normally distributed both overall and by quartile of attention switching (Figure). In addition, we evaluated variability in the mean number of minutes of inbox work averaged across all physicians among the 21 medical centers studied. In medical centers with a medium inbox work duration on workdays, the mean (SD) was 63.6 (18.1) minutes. Two medical centers were statistically higher than the mean, with a mean (SD) of 74.1 (20.5) minutes (*P* < .001), while 2 were lower than the mean, with a mean (SD) of 54.5 (14.9) minutes (*P* < .001).

**Figure.**
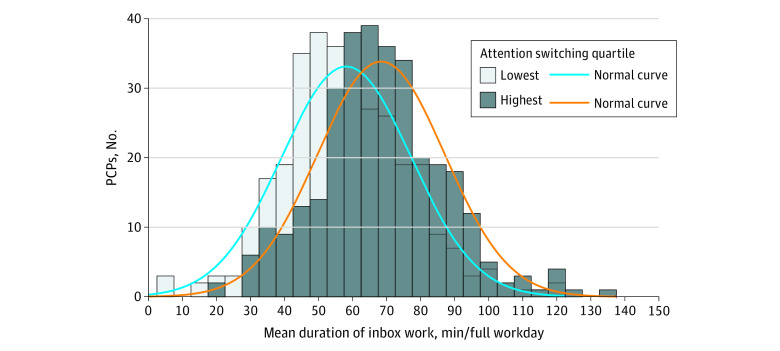
Distribution of Primary Care Physicians (PCPs) by Inbox Work Duration Attention switches are shown for PCPs in the highest vs lowest quartile.

In the final multivariable model for inbox work duration, higher quantities of inbox messages of all 4 types were associated with more minutes of inbox work per workday (eTable 2 in the Supplement). Each additional patient secure message beyond the reference value was associated with 0.151 (95% CI, 0.085-0.217) additional minutes, each additional results message was associated with 0.338 (95% CI, 0.272-0.404) additional minutes, each additional request message was associated with 0.101 (95% CI, 0.041-0.161) additional minutes, and each additional administrative message was associated with 0.179 (95% CI, 0.093-0.265) additional minutes. Having a patient panel with a higher percentage of patients who initiated a secure message during the study period was also associated with higher inbox work duration (0.386 minutes per percentage increase [95% CI, 0.026-0.745]). In addition, being a female physician (1.827 minutes per workday [95% CI 0.378-3.276]), older physician age (0.199 minutes per workday per year of age [95% CI, 0.092-0.307]), having fewer years with the medical group (0.273 minutes per year [95% CI, 0.139-0.406]), having a panel with a higher percentage of Asian patients (0.075 minutes per percentage increase [95% CI, 0.016-0.133]), working at a medical center with high inbox work duration among all physicians (4.161 minutes [95% CI, 2.029-6.292]), and more attention switching (0.373 minutes per switch [95% CI, 0.328-0.419]) were associated with higher inbox work duration. In contrast, having a higher number of work segments per message (−0.119 minutes per work segment [95% CI, −0.148 to −0.090]) and working at a medical center with low inbox work duration among all physicians (−2.730 minutes, [95% CI, −5.205 to −0.255]) were associated with lower inbox work duration. The final model explained most of the variability in the outcome (*R*^2^ = 0.66).

Table 4 illustrates the association of individual factors with the estimated minutes of inbox work. To characterize the effect size of each factor, we varied each one from lower to higher values (for continuous variables, the 25th and 75th percentiles) while holding all other variables at their reference values. The factors associated with the greatest variation in inbox work duration were working at a medical center with low or high mean inbox work duration (making a difference of 6.9 minutes between low and high), the quantity of messages about results (difference, 5.6 minutes), and the number of attention switches involving the inbox (difference, 10.2 minutes). Physician and patient panel characteristics explained less of the variability in inbox work duration.

**Table 4.** Variation Among Primary Care Physicians in Electronic Inbox Work Duration as Factors Are Varied From Low to High Values

Factor^a^	Value			Estimated No. of minutes of inbox work^c^		Additional No. of min of inbox work as factor is varied from low to high
	Reference^b^	25th Percentile or low	75th Percentile or high	25th Percentile or low	75th Percentile or high	
Message quantity, mean No. per workday						
Patient messages	29.0	18.9	36.8	62.0	64.7	2.7
Results	35.1	26.2	42.6	60.5	66.1	5.6
Requests	36.3	24.5	45.8	62.4	64.5	2.2
Informational	23.2	17.2	28.2	62.5	64.5	2.0
Unique messages per day, mean^d^	47.6	37.5	57.0	62.7	64.4	1.7
Patient panel characteristics						
Patients who initiated >1 secure message during the month, %	11.8	9.6	13.8	62.7	64.3	1.6
Patients with Asian race/ethnicity, %	19.7	8.1	26.7	62.7	63.9	1.4
Medical center categorized daily inbox work duration	Medium	Low	High	60.8	67.7	6.9
Other factors						
Attention switches with the electronic inbox, mean	79.4	65.1	92.5	58.2	68.5	10.2
Work segments per 100 unique messages, mean^d^	176	152	191	66.4	61.8	−4.6

^a^ The model was adjusted for physician age (2.6 more minutes when varied from the 25th to the 75th percentile), sex (1.8 more minutes for females than males), and years with the medical group (3.3 fewer minutes when varied from the 25th to the 75th percentile). See eTable 2 in the Supplement for the detailed estimates, 95% CIs, and *P* values from this model.

^b^ Reference value used in final multivariable model. When all values were set at the reference value, the physician was estimated to spend 63.6 minutes on inbox work in a full workday.

^c^ Per 24-hour period that included a workday. Estimated number is based on adjusted means from the final multivariable model. All variables in the table and in footnote a were significant at *P* < .05.

^d^ A unique message is a message opened for the first time during the month.

Physicians with a higher number of discrete work segments per unique message had lower estimated inbox work duration (61.8 minutes for the 75th percentile of work segments per message vs 66.4 minutes for the 25th percentile). In other words, this analysis did not find evidence that physicians whose work on inbox messages tended to be broken into multiple segments had higher inbox work duration; in fact, the reverse was true.

## Discussion

### Major Findings

Primary care physicians in this study made nearly 80 attention switches involving the electronic inbox on an average workday. Message quantity was a key correlate of both attention switching and inbox work duration. High inbox work duration was also associated with having a higher percentage of panel patients initiating messages and with working at a medical center with a high mean PCP inbox work duration. These findings suggest that interventions to assist PCPs with message quantity might help reduce both attention switching and inbox work duration.

### Context and Interpretation

To our knowledge, this is the first study to describe attention switching with the electronic inbox in medical practice. Attention switching is important whether due to exogenous events (the source of most interruptions) or endogenous factors. Psychological research on shifting attention among tasks has shown that it causes decrements in performance, including longer time required to complete each task and lower accuracy in completing the tasks.^18^ Studies in health care show that attention switching, both due to endogenous and exogenous factors, is similarly associated with lower performance^19^ and higher stress.^20^

Message quantity was the dominant factor associated with both attention switching and inbox work duration in our setting. Physician characteristics and patient panel size and demographics had far less association with attention switching and inbox work duration. This finding demonstrates that physicians multitask based on the context, rather than based on individual differences. Our finding that working at a medical center with high or low mean inbox work duration was independently associated with an individual physician’s inbox work duration suggests that some medical centers may have adopted processes that enhance the efficiency of inbox work for all PCPs in those locations. This result is consistent with a prior qualitative study that found that some departments in this setting had approaches to aid PCPs with inbox management, such as teams to help with messages or scheduled time for inbox management.^2^

Our observation that message quantity had a greater association than individual physician factors with attention switching has important implications for the design of structures to support PCPs. Studies in medicine suggest that interruptions may increase the likelihood of errors,^11,12^ consistent with the psychological literature showing that attempting to perform more than one task at a time imposes a cognitive burden.^21,22^ Primary care physicians attending to multiple competing demands may be unable to avoid a certain amount of task switching, similar to professionals in fields including the military and aviation. Other professions use training on effective task-switching skills to ease cognitive load and reduce the risk of error.^23,24^

Our analyses of inbox work duration, which has been suggested as a key metric for practice efficiency,^25^ included factors similar to but more extensive than those in previous studies.^26,27^ We observed that attention switching and inbox work duration were strongly associated, but this study’s design did not enable us to determine whether one factor caused the other. Thus, we constructed a variable that measured a physician’s propensity to not finish a message in one sitting. This led to the unexpected and potentially useful finding that inbox work duration was inversely associated with the mean number of work segments per message, even after controlling for multiple other factors. This finding signifies that work patterns that involve opening a message multiple times do not necessarily cause inefficiency. Future studies could use qualitative or time-motion methods to evaluate approaches associated with inbox work efficiency.

### Limitations

This study has some limitations. The medical group we studied is among the largest in the United States and has more than a decade of experience with its EHR. Its high level of integration may result in PCPs having higher quantities of electronic messages from specialist physicians, the pharmacy, and others than PCPs in less-integrated settings. Patients are encouraged to use EHR portal messages to communicate with their PCPs, while, in contrast, the group tries to minimize administrative messages. The generalizability of these findings to smaller or less integrated practices should be evaluated with these factors in mind.

In this study, the measurement of attention switching focused only on switches in and out of electronic inbox work. Preliminary analyses for this study suggested that attention switches involving other parts of the EHR were even more common than those involving the electronic inbox. Because we used EHR access log data to observe attention switching, we could not distinguish which switches represented external interruptions compared with self-guided changes in task. In addition, we did not analyze window switching (ie, switches between screens while working on an inbox message or other task). Window switching deserves further research.^27^

The duration of inbox work we observed was similar to that in other studies,^8,26^ suggesting that our approach was generally valid. However, our definitions of duration and attention switching variables relied on educated inferences that were inherently imperfect. For example, we allotted 120 seconds after a secure patient message was opened for the physician to type a reply and to take another action in the EHR before we classified it as non-EHR time. If a physician spent more than 120 seconds typing, we would have misclassified this type of event. These limitations likely generated a conservative estimate of time spent on inbox work.

## Conclusions

These findings suggest that attention switching with the electronic inbox is frequent among PCPs regardless of experience level and patient panel characteristics. Reductions in both attention switching and inbox work duration may be most amenable to measures that assist PCPs with message quantity.

## Notes

Supplement. **eFigure.** Conceptual Model Depicting Factors Studied as Potential Predictors of Attention Switching and Inbox Work Duration **eTable 1.** Multivariable Models of Factors Associated With Attention Switching Among Adult Primary Care Physicians, The Permanente Medical Group, Kaiser Permanente Northern California **eTable 2.** Multivariable Models of Factors Associated With Inbox Work Duration Among Adult Primary Care Physicians, The Permanente Medical Group, Kaiser Permanente Northern California
