# Supplementary material for: Evaluation of Attention Switching and Duration of Electronic Inbox Work Among Primary Care Physicians
Source: JAMA Netw Open. 2021 Jan 21;4(1):e2031856. doi: 10.1001/jamanetworkopen.2020.31856 (PMC7821028; doi:10.1001/jamanetworkopen.2020.31856)
Supplement: Supplement. — eFigure. Conceptual Model Depicting Factors Studied as Potential Predictors of Attention Switching and Inbox Work Duration eTable 1. Multivariable Models of Factors Associated With Attention Switching Among Adult Primary Care Physicians, The Permanente Medical Group, Kaiser Permanente Northern California eTable 2. Multivariable Models of Factors Associated With Inbox Work Duration Among Adult Primary Care Physicians, The Permanente Medical Group, Kaiser Permanente Northern California [file jamanetwopen-e2031856-s001.pdf]

Supplementary Online Content

Lieu TA, Warton EM, East JA, et al. Evaluation of attention switching and duration of electronic inbox work among primary care physicians. *JAMA Netw Open.* 2020;3(12):e2031856. doi:10.1001/jamanetworkopen.2020.31856

eFigure. Conceptual Model Depicting Factors Studied as Potential Predictors of Attention Switching and Inbox Work Duration

eTable 1. Multivariable Models of Factors Associated With Attention Switching Among Adult Primary Care Physicians, The Permanente Medical Group, Kaiser Permanente Northern California

eTable 2. Multivariable Models of Factors Associated With Inbox Work Duration Among Adult Primary Care Physicians, The Permanente Medical Group, Kaiser Permanente Northern California

This supplementary material has been provided by the authors to give readers additional information about their work.

© 2021 Lieu TA et al. *JAMA Network Open.*

eFigure. Conceptual Model Depicting Factors Studied as Potential Predictors of Attention Switching and Inbox Work Duration
The dashed arrows and ?s between attention switching and inbox work duration denote the possibility that either variable might be a determinant of the other, or that the relationship might be bidirectional.

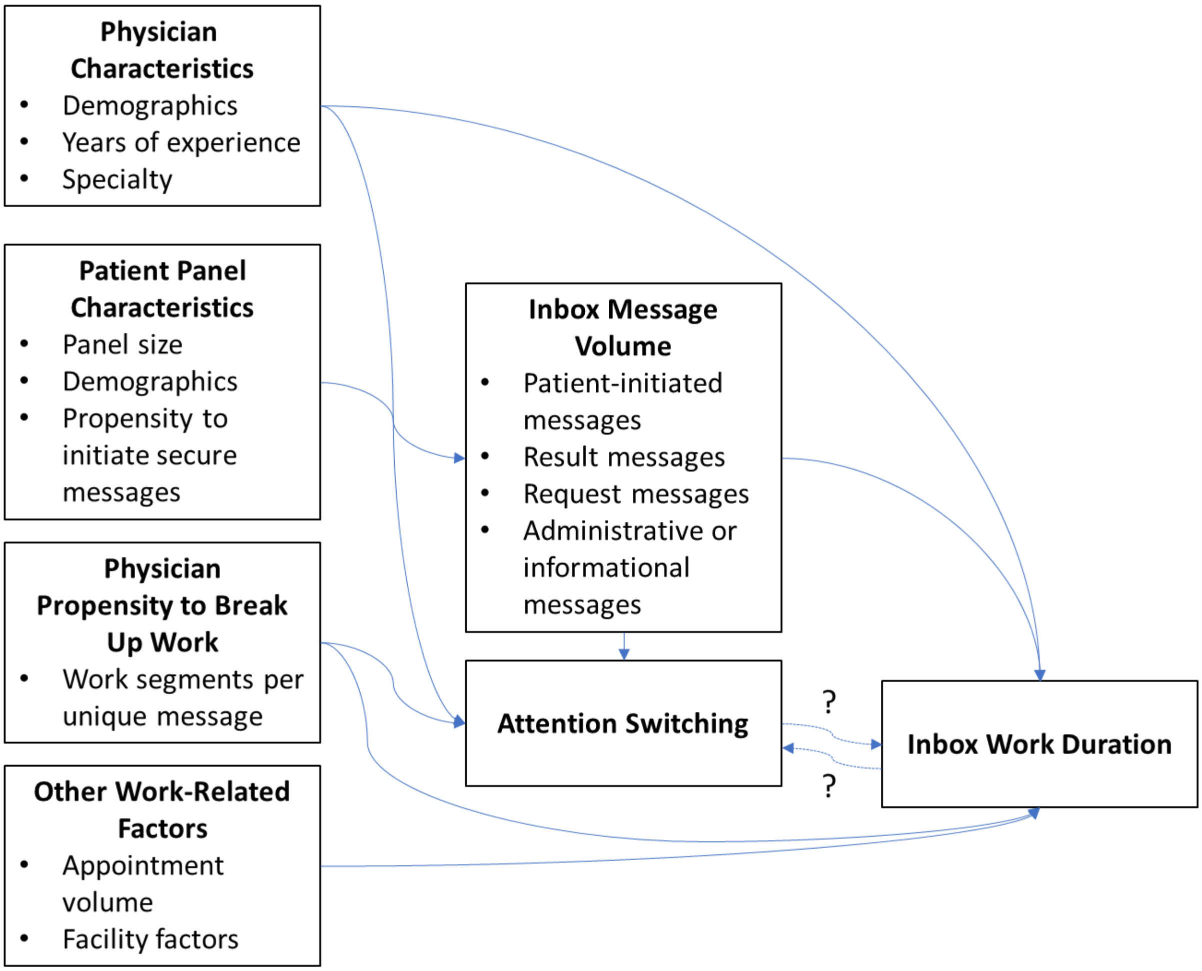

eTable 1. Multivariable Models of Factors Associated With Attention Switching Among Adult Primary Care Physicians, The Permanente Medical Group, Kaiser Permanente Northern California

Model 1 included physician demographics, panel size and appointment quantity; Model 3 added patient panel demographics and medical center factors to Model 1; and Model 6 added electronic inbox message factors to Model 3. The "estimates" represent beta-coefficients from the model. These indicate the number of additional switches that occur with each unit of change as a given factor is varied from the reference point or group. Numbers in bold denote factors that were significant at p<.05 in the given model.

**Variable**	**Model 1** (R^2 = 0.061)			**Model 3** (R^2=0.188)			**Model 6** (R^2=0.682)			
	Estimate	LCL	UCL	Estimate	LCL	UCL	Estimate	LCL	UCL	*P* Value
**Physician factors**										
Female	**3.078**	**0.659**	**5.497**	**3.078**	**0.659**	**5.497**	**-2.798**	**-4.416**	**-1.179**	<.001
Age (years, mean-centered)	**-0.380**	**-0.573**	**-0.187**	**-0.380**	**-0.573**	**-0.187**	**-0.124**	**-0.245**	**-0.003**	0.04
Years with this medical group	**0.470**	**0.254**	**0.686**	-0.089	-0.324	0.146	0.117	-0.033	0.267	0.13
Family medicine specialty[a]	-2.163	-4.638	0.312	**-2.897**	**-5.328**	**-0.465**	-0.363	-1.907	1.181	0.64
**Patient panel factors**										
Panel size	**0.007**	**0.004**	**0.010**	**0.011**	**0.007**	**0.014**	**0.003**	**0.000**	**0.005**	0.02
Total monthly appointments	0.008	-0.005	0.021	-0.005	-0.017	0.008	-0.009	-0.018	0.000	0.06
Percent of panel patients who were:										
Hispanic				**0.188**	**0.052**	**0.325**	0.003	-0.086	0.092	0.95
Black				0.152	-0.042	0.346	0.030	-0.094	0.155	0.64
Asian				0.090	-0.007	0.187	-0.012	-0.077	0.054	0.73
Other				-0.066	-0.529	0.397	-0.138	-0.431	0.155	0.36
≥65 years old				**0.508**	**0.308**	**0.709**	**0.144**	**0.009**	**0.278**	0.04
**Medical center average inbox work duration**										
High				7.589	3.880	11.298	0.153	-2.247	2.553	0.90
Low				-7.038	-11.257	-2.819	-2.703	-5.475	0.068	0.06

© 2021 Lieu TA et al. *JAMA Network Open.*

**Variable**	**Model 1** (R^2 = 0.061)			**Model 3** (R^2=0.188)			**Model 6** (R^2=0.682)			
	Estimate	LCL	UCL	Estimate	LCL	UCL	Estimate	LCL	UCL	*P* Value
**Inbox message factors**										
Percent of panel initiating ≥1 secure message during the month				**2.023**	**1.486**	**2.561**	0.036	-0.367	0.439	0.86
Mean daily number of:										
Patient secure messages							**0.289**	**0.217**	**0.362**	<.001
Results messages							**0.203**	**0.127**	**0.278**	<.001
Request messages							**0.190**	**0.124**	**0.257**	<.001
Administrative messages							**0.262**	**0.166**	**0.358**	<.001
Mean unique messages per day, over 31 days							0.015	-0.070	0.100	0.73
Mean daily minutes of inbox work							**0.468**	**0.411**	**0.524**	<.001
Mean work segments per 100 unique messages							**0.129**	**0.097**	**0.162**	<.001

LCL, lower 95% confidence limit; UCL, upper 95% confidence limit

[a]The reference group was internal medicine.

© 2021 Lieu TA et al. *JAMA Network Open.*

eTable 2. Multivariable Models of Factors Associated With Inbox Work Duration Among Adult Primary Care Physicians, The Permanente Medical Group, Kaiser Permanente Northern California

Model 1 included physician demographics, panel size and appointment quantity; Model 3 added patient panel demographics and medical center factors to Model 1; and Model 6 added electronic inbox message factors to Model 3. The "estimates" represent beta-coefficients from the model. These indicate the number of additional minutes of work that occur with each unit of change as a given factor is varied from the reference point or group. Numbers in bold denote factors that were significant at p<.05 in the given model.

**Variable**	**Model 1** (R^2=0.097)			**Model 3** (R^2=0.277)			**Model 6** (R^2=0.656)			
	Estimate	LCL	UCL	Estimate	LCL	UCL	Estimate	LCL	UCL	*P* Value
**Physician factors**										
Female	**6.568**	**4.529**	**8.608**	**3.164**	**1.111**	**5.218**	**1.827**	**0.378**	**3.276**	0.01
Age (years, mean-centered)	-0.041	-0.204	0.122	-0.040	-0.194	0.114	**0.199**	**0.092**	**0.307**	<.001
Years with this medical group	**0.270**	**0.088**	**0.452**	**-0.303**	**-0.495**	**-0.112**	**-0.273**	**-0.406**	**-0.139**	<.001
Family medicine specialty[a]	-1.112	-3.199	0.975	-1.760	-3.737	0.217	-0.682	-2.062	0.697	0.33
**Patient panel factors**										
Panel size	**0.008**	**0.006**	**0.011**	**0.012**	**0.009**	**0.014**	0.000	-0.002	0.002	0.91
Total monthly appointments	0.010	-0.001	0.021	-0.003	-0.013	0.008	-0.006	-0.014	0.002	0.16
Percent of panel patients who were:										
Hispanic				**0.217**	**0.107**	**0.328**	0.016	-0.064	0.096	0.69
Black				**0.234**	**0.077**	**0.392**	0.107	-0.004	0.218	0.06
Asian				**0.154**	**0.075**	**0.233**	**0.075**	**0.016**	**0.133**	0.01
Other				-0.135	-0.512	0.241	-0.024	-0.287	0.238	0.86
≥65 years old				**0.504**	**0.341**	**0.667**	0.023	-0.097	0.144	0.70
**Medical center average inbox work duration**										
High				**8.858**	**5.842**	**11.874**	**4.161**	**2.029**	**6.292**	<.001
Low				**-8.325**	**-11.756**	**-4.894**	**-2.730**	**-5.205**	**-0.255**	0.03

© 2021 Lieu TA et al. *JAMA Network Open.*

**Variable**	**Model 1** (R^2=0.097)			**Model 3** (R^2=0.277)			**Model 6** (R^2=0.656)			
	Estimate	LCL	UCL	Estimate	LCL	UCL	Estimate	LCL	UCL	*P* Value
**Inbox message factors**										
Percent of panel initiating ≥1 secure message during the month				**2.010**	**1.573**	**2.447**	**0.386**	**0.026**	**0.745**	0.04
Mean daily number of:										
Patient secure messages							**0.151**	**0.085**	**0.217**	<.001
Results messages							**0.338**	**0.272**	**0.404**	<.001
Request messages							**0.101**	**0.041**	**0.161**	<.001
Administrative messages							**0.179**	**0.093**	**0.265**	<.001
Mean unique messages per day, over 31 days							**0.089**	**0.013**	**0.165**	0.02
Mean attention switches into and out of the inbox							**0.373**	**0.328**	**0.419**	<.001
Mean work segments per 100 unique messages							**-0.119**	**-0.148**	**-0.090**	<.001

LCL, lower 95% confidence limit; UCL, upper 95% confidence limit

[a]The reference group was internal medicine.

© 2021 Lieu TA et al. *JAMA Network Open.*
